# Effect of neoadjuvant chemotherapy and its correlation with HPV status, EGFR, Her-2-neu, and GADD45 expression in oral squamous cell carcinoma

**DOI:** 10.1186/s12957-018-1308-7

**Published:** 2018-01-31

**Authors:** Manoj Pandey, Krishna Kiran Kannepali, Ruhi Dixit, Mohan Kumar

**Affiliations:** 1 Department of Surgical Oncology, Institute of Medical Sciences, Banaras Hindu University, Varanasi, 221 005 India; 2 Department of Surgery, Institute of Medical Sciences, Banaras Hindu University, Varanasi, 221 005 India; 3 Department of Pathology, Institute of Medical Sciences, Banaras Hindu University, Varanasi, 221 005 India

**Keywords:** Oral cancer, Buccal mucosa, Tongue, Alveolus, Survival, EGFR, Her-2-neu, GADD45, Human papilloma virus

## Abstract

**Background:**

Head and neck cancers are the commonest cancer in Southeast Asia. Despite being a surface cancer, it is associated with significant morbidity as despite early detection by the patients they often report for treatment late and hence are associated with poor prognosis. The role of neoadjuvant chemotherapy in head and neck cancer is still under evaluation; there is a large subgroup of population that does not respond to chemotherapy, and hence, most studies have failed to show any survival benefit. This study evaluated the role of neoadjuvant therapy with docetaxel and carboplatin in patients with oral cancer and correlated the response to human papilloma virus, EGFR1, EGFR2, and GADD45 expression.

**Methods:**

A total of 24 locally advanced, non-metastatic oral cancer patients were included in the study. Tumor biopsies were taken prior to the start of neoadjuvant therapy for expression of EGFR, Her-2-Neu, and GADD45 by immunohistochemistry and for HPV by PCR. The response was evaluated using Response Evaluation Criteria in Solid Tumors (RECIST) criteria after three cycles of chemotherapy. Statistical analysis was performed using correlation and Kaplan–Meier analysis; the difference in survival was calculated with log rank test.

**Results:**

A total of 21 male and 3 female with a mean age of 53.12 years were enrolled. Sixty-five percent of these received three cycles of chemotherapy. Five patients were positive for HPV 16 and none for HPV 18. Twenty-two of 24 patients showed GADD45 expression, 3 showed expression of Her-2-Neu while all 24 showed expression for EGFR1 protein. Two-year overall survival was 81%; GADD45 expressions were found to significantly affect the overall and disease-free survival, while any of the other protein expression studied and HPV status was not significant.

**Conclusion:**

The result of the present study shows significant downgrading of the oral cancers with neoadjuvant chemotherapy suggesting its utility in borderline operable cases. However, the response of chemotherapy does not appear to be related to the expression of EGFR, Her-2-Neu, and GADD45 protein or presence of HPV. Bone involvement, perineural invasion, and GADD45 expression significantly predict OS and DFS. All patients who did not express Gadd45 died before 2 years. Study with more subjects and longer follow-up should be carried out to elucidate this relation further.

## Background

Head and neck cancer squamous cell carcinoma (HNSCC) is the sixth most common cancer with an annual incidence of approximately 4,00,000 worldwide. Two third of the global incidence is reported from developing countries, of which one third is from India. The survival rate of HNSCC is poor, and the disease has a poor prognosis. The treatment of early cancer is surgery; however, in later stages, the chemotherapy (CTh) and radiotherapy [1] are preferred. Several new chemotherapeutic agents including taxanes and platinums have been used as first-line treatment in head and neck cancer; however, the response rate with this doublet or triplet combination ranges from 40 to 60%.

Human papilloma virus (HPV) has been reported to be a causative factor in a subset of HNSCC that usually has a favorable prognosis [2]. High-risk HPV (HPV 16, 18, and 33)-related HNSCC is associated with certain sexual behavior, such as oral sex and increasing numbers of sexual partners, and these tumors usually lack association with smoking and alcohol [3]. Several studies and meta-analysis have confirmed that HPV-related HNSCC behaves differently from HPV-negative SCC and is associated with better treatment response to chemotherapy and radiation [4, 5].

Growth arrest and DNA damage repair (GADD45) gene plays important and diverse roles in the regulation of cell-cycle [6]. Gadd 45 protein interacts with several regulatory pathways [7]. GADD45 promoter hypermethylation is frequently detected in tumor cell lines, including 85% of non-Hodgkin’s lymphoma, 50% of Hodgkin lymphoma, 73% of nasopharngeal, 50% of cervical, 29% of esophageal, and 40% of lung carcinomas [7]. The role of GADD45a as a potential therapeutic target has become a matter of interest due to the fact that it is upregulated on docetaxel treatment. Because of the various functions of GADD 45 in cell-cycle control and its dysregulation in cancer, chemotherapeutic and molecular agents targeting GADD45 proteins may serve as novel therapeutic interventions in cancer treatment. Ramachandran et al. [8] described GADD45a novel potential therapeutic target which has been highlighted by the fact that it is upregulated on docetaxel treatment and may contribute to docetaxel mediated cytotoxicity of prostate cancer. In other study, authors found an increase in the GADD45A and GADD45B in docetaxel- and estramustine-treated prostate cancer cells which suggest their affects in the induction of apoptosis [9].

EGFR family includes EGFR, Her-2-neu (c-erb-B-2), her-3 (c-erb-B-3), and her 4 (c-erb-B-4) [10]. EGFR and Her-2-neu is a transmembrane tyrosine kinase receptor with intrinsic tyrosine kinase activity [10]. Several studies have found correlation between EGFR overexpression and survival in oral SCC and HNSCC [11, 12]. Their expression and association with therapeutic targets have been demonstrated.

The big challenge is to identify which treatment is best suited for the individual patient by studying tumor biology at a molecular level and identifying biomarkers for predicting the response to therapy. Aim of the study was to study the effect of neoadjuvant chemotherapy with docetaxel and carboplatin in locally advanced non-metaplastic oral squamous cell carcinomas and to correlate the tumor response and survival with HPV status, EGFR, Her-2-neu, and GADD45 expression.

## Methods

### Patients

A total of 24 patients attending the surgical oncology outpatient between December 2009 to August 2011 with histologically proven locally advanced, borderline operable oral squamous cell cancers (OSCC) were included in the study. Those who had a history of severe hypersensitivity reaction to docetaxel were excluded. The study was approved by the Institute Ethics Committee. Informed consent was taken from all the patients prior to recruitment and collection of specimen. Tissue was stored at − 80 °C till analysis.

Biopsy was taken to study the HPV positivity, EGFR, GADD45, and Her-2-neu expression. All patients underwent neoadjuvant chemotherapy (NACT) with docetaxel 80 mg/m^2^ and carboplatin 300 mg/m^2^ on day 1 as a three weekly regimen, and response of therapy was correlated to HPV positivity, EGFR, GADD45, and Her-2-neu expression. Responses were evaluated by clinical examination and post therapy CT scan by Response Evaluation Criteria in Solid Tumors (RECIST) criteria (complete response, partial response, progressive disease, and stable disease).

### Expression analysis by PCR

#### DNA isolation

DNA isolation was carried out by phenol-chloroform-isoamyl alcohol method.

#### PCR

Consensus primers were used to amplify the HPV16 and HPV18, i.e., MY09/11 and GP05/06. Primer sequence for MY09/11 was MY09—5′-CGTCCMARRGGAWACTGATC-3′ and MY11—5′-GCMCAGGGWCTATAAYAATGG-3′ and product size was 450 bp for HPV 16, while primer sequence for GP05/06 was GP-05—5′-TTTGTTACTGTGGTAGATACYAC-3′ and GP-06—5′-GAAAAATAAACTGTAAATCATATTC-3′ and product size was 140 bp for HPV 18. PCR was conducted in Biometra Thermal Cycler. Reaction mixture contains micromole of oligonucleotide, 2× PCR buffer, 100 ng of DNA template, and nuclease-free water. Hot start PCR program for both primers are initial denaturation at 95 °C for 5 min, denaturation at 94 °C for 45 s, annealing at 47.5 °C for 45 s, extension at 72 °C for 45 s followed by 40 cycles, and final extension at 72 °C for 7 min. Agarose gel electrophoresis was carried out to check the PCR amplification.

### Immunohistochemistry

Immunohistochemistry was performed on formalin-fixed paraffin blocks which cut at a thickness of 2 μm. These sections were deparaffinized in xylene followed by hydration in a graded series of alcohols. Sections were left under running water for 15 min. Endogenous peroxidase activity was blocked by incubation in 3% hydrogen peroxide for 30 min at room temperature. After rinsing in TBS buffer (pH 7.4) for 30 min, the sections were incubated with primary antibody at 4 °C overnight. The following primary antibodies were used: EGFR (SantaCruz Biotechnology, Santa Cruz, CA), GADD45A (Abcam, Cambridge, MA), and Her-2-neu (Biogenex). Slides were then washed again in TBS. Secondary antibody was added, and slides were washed again after 30 min. The labeled antigen–antibody complexes were visualized as brown pigments via a standard DAB (Zhongshan Jinqiao Co., Beijing, China) protocol. DAB was added and kept for 10 min until brown color developed. Slides were washed in running water, and counterstaining was done with hematoxylin. Slides were then blotted, dried, and mounted.

### Statistical analysis

Correlations were performed by using Pearson’s and Spearman’s correlation coefficient with two-tailed significance. Survival was compared using Kaplan–Meier method, and the difference in survival was compared by using log rank test. *p* < 0.05 was considered as statistically significant. Analyses were performed with SPSS software (version 13.0; SPSS Inc., Chicago, IL, USA).

## Results

### Clinicopathological features

Out of 24 patients enrolled, 21 (87.5%) and 3 (12.5%) were males and females respectively. Mean age of the study population was 53.12 years (range 25–72 years). Eighty-eight percent of the patients had the habit of tobacco and alcohol, and only 2% patient did not have the habit of tobacco consumption in any form. Most of the cases were of carcinoma of buccal mucosa (29%), lower alveolus (25%), and tongue (25%). The most common symptoms among the patients were ulcer (96%) and pain (71%). Other symptoms include dyspahgia (17%), loosening of teeth (17%), bleeding (13%), trismus (13%), and dysarthia (8%). The primary growth was of ulcero-proliferative type in 50% patients and of infiltrative type in 50% cases. Eighteen out of 24 patients had extension to the adjacent skin, muscle, or bone either alone or combination of more than one. Majority of the patients had T4 disease (19; 79%) while 13 (54%) had N2 disease (Table 1).

**Table 1:** Clinicopathological characteristics of oral squamous cell carcinoma

Parameters	Frequency
1. Gender distribution	
Male	21 (87.5%)
Female	3 (12.5%)
2. Age distribution	
Mean	53.12 years
3. Precancerous lesion	
No precancerous lesion	22 (92%)
Leucoplakia	2 (8%)
4. Habit of tobacco chewing and alcohol addiction	
Single habit present	21 (88%)
Absent	2 (8%)
Tobacco + alcohol	1 (4%)
5. Site of distribution	
Buccal mucosa	7 (29%)
Lower alveolus	6 (25%)
Tongue	6 (25%)
Retromolar trigone	3 (13%)
Lower lip	1 (4%)
GB sulcus	1 (4%)
6. TNM stage	
T2	3 (13%)
T3	2 (8%)
T4a	19 (79%)
N0	3 (13%)
N1	8 (33%)
N2b	11 (46%)
N2c	2 (8%)
M0	0
7. Stage grouping	
III	3 (13%)
IVA	21 (87%)

### HPV status, GADD-45, EGFR, and Her-2-neu expression

**Fig. 1:**
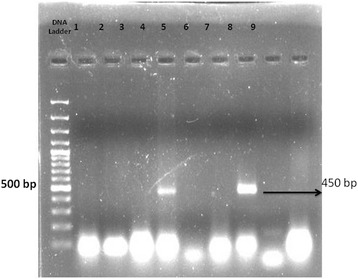
Image showing the HPV16 status in oral squamous cell carcinoma

**Fig. 2:**
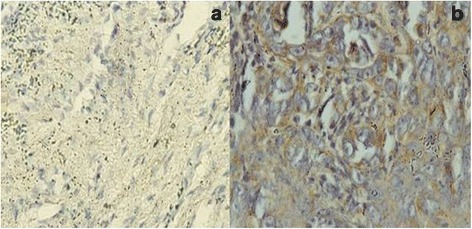
Immunohistochemistry for GADD45: **a** Grade 1 positive cytoplasmic staining and **b** grade 3 positive nuclear and cytoplasmic staining in oral squamous cell carcinoma

**Fig. 3:**
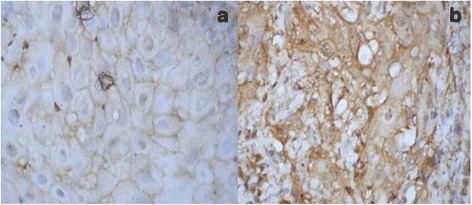
Immunohistochemistry for EGFR: **a** weak positive staining and **b** strong positive staining in oral squamous cell carcinoma

**Fig. 4:**
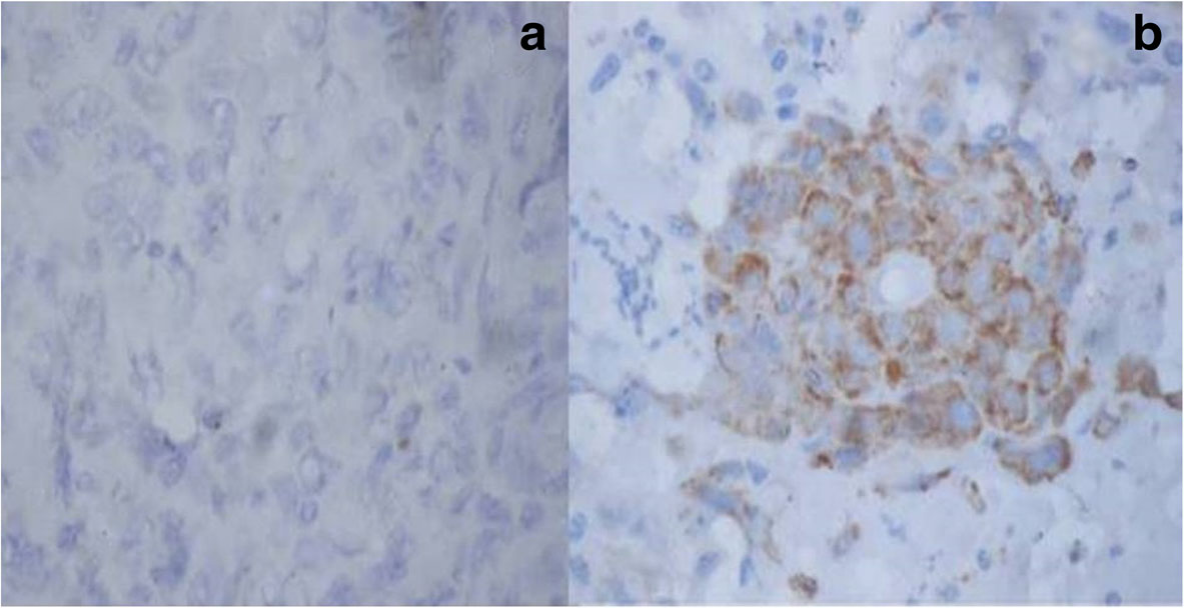
Immunohistochemistry for Her-2-neu: **a** negative staining and **b** positive staining in oral squamous cell carcinoma

PCR for HPV was performed on the tumor samples in all the 24 patients. Out of 24 samples, 5 were found to be positive for HPV16 type whereas HPV18 was not found in any of the cases (Fig. 1). GADD45 expression was shown in 22 (92%) out of 24 patients. All the patients had cytoplasmic expression of GADD45 (Fig. 2). There was only one patient which had additional staining also. Grade I staining was present in 2 (6%), grade II in 15 (63%), and grade III in 5 (21%) patients (Fig. 2). EGFR was expressed in all the cases (100%). EGFR showed grade I staining in 6 (25%), grade II in 8 (33%), and grade III in 10 (42%) patients (Fig. 3). Out of 24 samples, only 3 (12.5%) samples expressed Her-2-neu. The expression was mild in all the 3 cases (Fig. 4).

Out of 24 patients, 2 patients received 2 cycles, 15 received 3, 3 patients received 5, and only 1 patient received 6 cycles of neoadjuvant chemotherapy with docetaxel (80 mg/m^2^) and carboplatin (300 mg/m^2^). The response to CTh was assessed according to RECIST criteria. One (4%), 11 (46%), 8 (33%), and 4 (17%) patients had complete, partial, stable, and progressive response on CTh respectively. The overall response rate was 50%. The response of NACT on the size of tumor was compared before and after CTh. The mean length and breadth of the lesions before CTh were 4.18 and 2.8 cm while post CTh, it was 3.22 and 2.6 cm respectively.

### Correlation between markers, T and N stage, performance status, and response

Her-2-neu was expressed in tumors with high EGFR expression. The correlation coefficient (*r*) was 0.4, and *p* value is 0.048. There was no statistically significant correlation between CTh response and EGFR, Her-2-neu, and GADD45 expression, and HPV status (Table 2). Patients with HPV-positive tumors have a better performance status (*r* = + 0.39, *p* < 0.05). Patients with better performance status have a better response to CTh (*r* = + 0.52, *p* < 0.05). No significant correlation was observed between T, N, and composite stage and the markers under study (Table 2).

**Table 2:** Expression status of EGFR, GADD-45, EGFR, and Her-2-neu and response rate of chemotherapy on oral cancer patients

	GADD45	HPV status	EGFR status	Her-2-neu	Response
GADD45					
r	1	0.292	− 0.047	− 0.076	− 0.263
*p*	–	0.187	0.834	0.735	0.236
*N*	22	22	22	22	22
HPV status					
r	0.292	1	− 0.095	− 0.116	0.103
*p*	0.187	–	0.659	0.508	0.633
*N*	22	24	24	24	24
EGFR status					
r	− 0.047	− 0.095	1	0.408	0.321
*p*	0.834	0.659	–	0.048	0.126
*N*	22	24	24	24	24
Her-2-neu					
r	− 0.076	0.116	0.408	1	0.378
*p*	0.735	0.588	0.048	–	0.069
*N*	22	24	24	24	24
Response					
r	− 0.263	0.103	0.321	0.378	1
*p*	0.236	0.633	0.126	0.064	–
*N*	22	24	24	24	24

*r* correlation coefficient, *p* value, *N* no of positive patients

### Two-year survival

The 2-year overall survival (OS) was 81%. There were 2 females on follow-up, and both of them died within 1 year. In males, the 2-year overall survival was 89% (*p* = 0.00). Two-year overall survival by GADD45 expression grade was significantly different. Of the 24 patients, only 2 did not express GADD45 and both of them died at 2 years. There was no statistical significant difference in 2-year overall survival with relation to HPV status, EGFR, and Her-2-neu expression (Table 3).

**Table 3:** Association of the 2-year overall survival and disease-free survival with different clinicopathological variables and biomarkers

	2-year overall survival (OS) (%)	*p* value	2-year disease-free survival (DSF) (%)	*p* value
Gender				
Male	89.5	0.00*	73.7	0.00*
Female	0		0	
T stage				
T2	100	0.541	33.3	0.18
T3	100		50	
T4	75		75	
N stage				
N0	100	0.738	100	0.422
N1	75		50	
N2	81.8		72.7	
Skin involvement				
Present	100	0.242	100	0.095
Absent	75		56.2	
Bone involvement				
Present	90	0.282	90	0.023*
Absent	72.7		45.5	
GADD45				
0	0	0.005*	0	0.012*
1	100		100	
2	83.3		58.3	
3	100		100	
HPV status				
Positive	100	0.234	100	0.077**
Negative	75		56.2	
EGFR grading				
1	83.3	0.566	83.3	0.518
2	60		40	
3	90		70	
Her-2-neu				
Positive	100	0.392	66.7	0.933
Negative	77.8		66.7	
Pathological lymph node involvement				
Present	81.8	0.899	63.7	0.47
Absent	80		70	
Vascular invasion				
Present	71.4	0.273	66.7	0.86
Absent	88.9		71.4	
Perineural invasion				
Present	57.1	0.05*	57.1	0.36
Absent	100		77.8	
Lymphatic invasion				
Present	71.4	0.273	66.7	0.86
Absent	88.9		71.4	

*statistically significant; **approaching statistical significance

### Disease-free survival

**Fig. 5:**
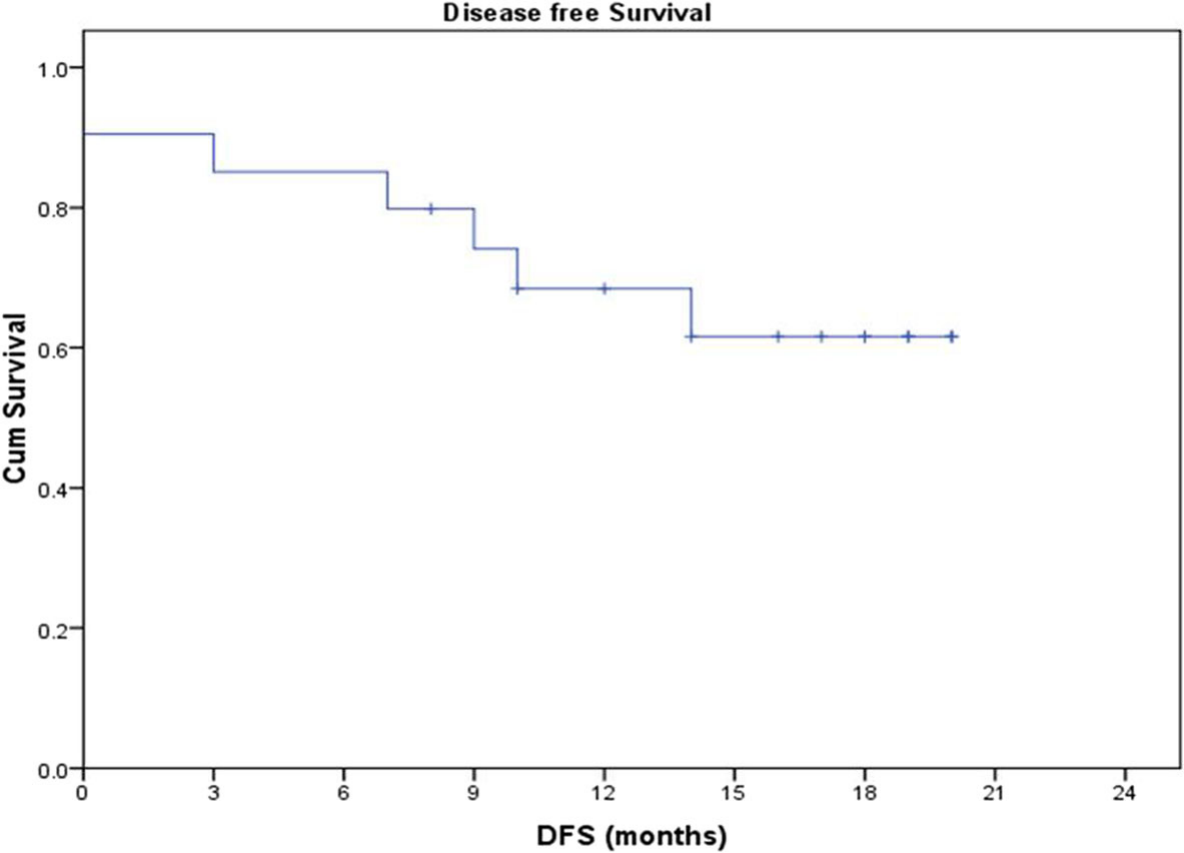
Disease-free survival

**Fig. 6:**
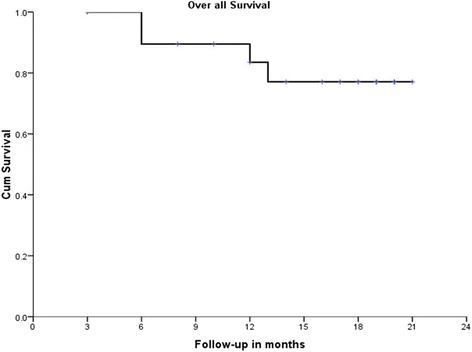
Overall survival

**Fig. 7:**
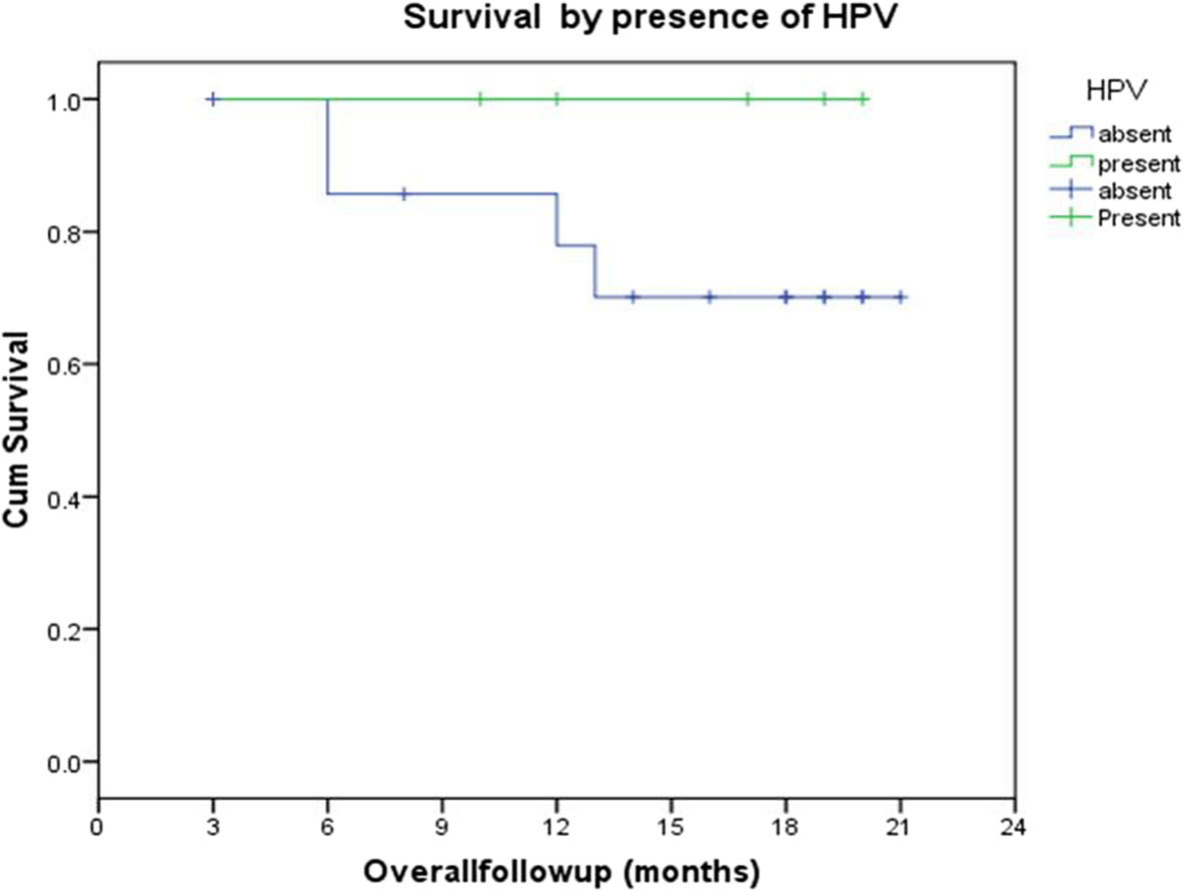
Survival by presence of HPV

The overall 2-year disease-free survival (DFS) in the study was 66.7% (Figs. 5 and 6). Those cases which did not express GADD45 had a 0% 2-year DFS while it was 100, 58.3, and 100% in grade 1, 2, and 3 staining respectively. The difference was statistically significant (*p* = 0.012); however, it was not consistent pattern. The 2-year DFS in HPV-positive patients was 100% and in HPV-negative patients, 56.2% (*p* = 0.077); the HPV-positive patients had better DFS; however, it did not reach statistical significance (Fig. 7). EGFR and Her-2-neu did not correlate significantly with 2-year DFS.

## Discussion

The treatment of squamous cell carcinoma of head and neck (HNSCC) has been the realm of surgeons and radiation oncologists. There has been a paradigm shift in the management of HNSCC over the last two decades, and now, chemotherapy (CTh) is an integral part of the management. Therefore, it is a big challenge to individualize the treatment. Thus, this study was done to identify predictors of response to CTh with docetaxel-based regimen in locally advanced oral squamous cell carcinoma and correlated the tumor response with HPV status, EGFR, Her-2-neu, and GADD45 expression. In our study, 46% of patients had partial response to CTh. The complete response rate (CRR) was 41.6%, and overall response rate (ORR) was 50%. Lip tumors responded best to CTh followed by GB sulcus tumors, 57% of buccal mucosa, 50% each of tongue and alveolus lesion responded to CTh. Colevas et al. reported a CRR of 61% and ORR of 100% using the TPFLS regimen in 23 patients with HNSCCC [13]. Their other study also reported a CRR of 63% and ORR of 93% in 30 patients with HNSCC [13] using the TPFl4 regime. While Posner et al. in 2001 got a CRR of 40% and ORR of 93% in 43 patients with HNSCC using the TPF regimen [14]. Watanabe et al. in 2004 [15] reported a CRR of 59% and ORR of 88% in 34 patients with HNSCC using the TPF regimen with a lower done of docetaxel. In the phase III TAX323 trial which used induction CT with TPF regimen followed by RT, the CRR was 8.5% and ORR was 68% [16, 17]. Albers et al. [18] recently reported an overall response rate of 86% and 3-year survival of 65% using the docetaxel-based chemotherapy in combination in locally advanced cancer wherein 30/45 of his patients were treated with curative intent. Though role of neoadjuvant chemotherapy in head and neck cancers is debatable, however, recent evidence suggests some possible role. Inhestern et al. [19] reported a 97% 2-year OS for responder compared to 76% in those who did not respond to TPF therapy. In all of the patients, the chemotherapy was followed by surgery and concurrent chemoradiation. However, all other studies have failed to show any improvement in the overall or disease-free survival, and some have even showed disease progression and operative complications in patients receiving neoadjuvant chemotherapy [20–23].

### Correlation of EGFR, Her-2-neu, and GADD45 expression and HPV status with response to NACT with docetaxel and survival

In our study, all the cases expressed EGFR while Her-2-neu was expressed in 3 (12.5%) out of 24 patients. Her-2-neu expression was associated with high EGFR expression (correlation coefficient *r* = 0.4; *p* = 0.048). Neither EGFR expression nor Her-2-neu expression was significantly associated with response to CTh with docetaxel (*r* = 0.321; *p* = 0.126 and *r* = 0.378; *p* = 0.69 for EGFR and Her-2-neu respectively) and with 2-year disease-free and overall survival. In an earlier study, EGFR was expressed in 79.4% of cases of recurrent HNSCC [24]. EGFR-positive tumors had a 3-year cause-specific survival of 27.2% after salvage surgery whereas EGFR-negative tumors had a 3-year cause-specific survival of 64.3% (*p* < 0.001) [24]. The EGFR expression alone had no significant impact on survival [24]. In another study by Shiraki et al., the overall EGFR expression was 39% and in T3 and T4 lesions, it was 37%. In the same study, co-expression of EGFR, p53, and cyclin D1 was associated with shortened survival [11]. Xia et al. observed that at the combine expression of EGFR, Her-2-neu and Her-3 is a stronger predictor of outcome in oral SCC than any individual family members of the EGFR family [12]. EGFR expression (87.5%) was not found to be associated with proliferation, apoptosis, angiogenesis, and lymphangiogenesis in oral SCC [25]. Chen et al. looked at the EGFR and Her-2-neu expression in oral SCC by enzyme immunoassay method. In their study, EGFR was overexpressed in 58% tumors and Her-2-neu in 41%. EGFR overexpression was significantly associated with TNM stage, lymph node involvement and extra capsular spread, and poor survival. Her-2-neu was not found to be associated with any of the above parameters [26].

GADD45 was expressed in 91.6% cases in the study. There was no significant association established between GADD45 expression and response to CTh with docetaxel and carboplatin (*r* = 0.263; *p* = 0.236). But a significant association was there between GADD45 expression and 2-year disease-free and 2-year overall survival. Those cases which did not express GADD45 had a poor 2-year overall survival of 0% compared to 81% when compared with positive cases (*p* = 0.005). There is no published report examining the role of GADD45 as a predictive marker of CT response or survival in LA HNSCC till date. In prostate cancer, upregulation of GADD45 levels has been associated with increased sensitivity to docetaxel in in vitro studies [8]. They have proposed that GADD45 was epigenetically repressed in prostate cancer and its upregulation may be a potential target for therapeutic strategies [8]. Another experimental study had concluded that tumors in GADD45 deficient mice show increased mutation frequency and increased susceptibility to ionizing radiation and to chemical carcinogens [27]. All our cases that expressed GADD45 did so before starting the CTh, and post therapy evaluation was not carried out in this study.

### HPV positivity and correlation with CTh response and survival

In our study, PCR for HPV 16 and 18 types were performed and only 5 (20.83%) was found to be HPV 16 type positive. Like our previous study, this study too failed to demonstrate any significant association between HPV status and response to NACT with docetaxel and carboplatin (*r* = 0.103; *p* = 0.633) [5]. The 2-year overall survival in HPV-positive patient was 100% and in HPV negative patients, 75%, and the difference was not significant (*p* = 0.234). The 2-year DFS in HPV-positive patients was 100% and in HPV negative patients were 56.2%, and the difference was almost approached statistically significant (*p* = 0.077).

The impact of HPV on cancer risk and overall survival in head and neck squamous cell carcinomas has been summarized in a meta-analysis, concluding that 21.95% of HNSCC patients are HPV positive and the prevalent genotype is HPV-16 (86.69%). A better survival in HPV-positive patients has been reported compared to HPV-negative cases [27]. Prevalence of HPV is reported to be 41% in oropharengeal cancers [28]. Fakhry et al. looked at the impact of HPV positivity on survival in stage 3 and 4 oropharengeal cancers and reported that the HPV-positive patients had higher response rate after induction CTh (*p* = 0.01) than HPV-negative patients and after chemoradiation treatment (*p* = 0.007) [28].

Another study also reported that presence of HPV was significantly correlated with a better survival in patients with oral squamous cell carcinoma [29]. Most of the studies have reported a role of HPV as a predictor to response to treatment though our study failed to do so. That may be due to small sample size. Studies with larger sample size may clarify their association better.

### Relation of survival with other parameters

In our study, 2-year OS and DFS were 81 and 66.7% respectively. Colevas et al. had got an 83% 2-year OS by using TPF regimen [30]. Some other authors also observed a 60% 2-year OS by using TPF regimen [31]. Others have also found a 2-year OS as 79, 60, 41, and 93% [15, 31–33]. Another group demonstrated a 3-year OS with the TPF regimen and got only 29.6 [34]. These results showed that the response rates have been variable. It appears that the genetic and molecular characteristics of the tumor do play a role; however, so far, no such markers for prediction of the tumor response have been identified.

## Conclusions

Our findings suggests that HPV status, EGFR, and GADD45 be assessed in OSCC as together they may help in predicting the survival of the patients; though GADD45 expression was found to predict survival and DFS, however, it did not predict the response to chemotherapy with docetaxel.
